# Connection, constraint, and coping: A qualitative study of experiences of loneliness during the COVID-19 lockdown in the UK

**DOI:** 10.1371/journal.pone.0258344

**Published:** 2021-10-13

**Authors:** Phoebe E. McKenna-Plumley, Lisa Graham-Wisener, Emma Berry, Jenny M. Groarke

**Affiliations:** Centre for Improving Health-Related Quality of Life, School of Psychology, Queen’s University Belfast, Belfast, United Kingdom; University of Bologna, ITALY

## Abstract

The COVID-19 pandemic has necessitated physical distancing which is expected to continue in some form for the foreseeable future. Physical distancing policies have increased reliance on digital forms of social connection and there are widespread concerns about social isolation and mental health in this context. This qualitative study sought to understand how loneliness was experienced during physical distancing in the initial national UK COVID-19 lockdown. Eight individuals who reported feeling lonely during the initial lockdown were interviewed in May 2020. Interviews were analysed using reflexive thematic analysis. Four main themes were identified: (1) Loss of in-person interaction causing loneliness, (2) Constrained freedom, (3) Challenging emotions, and (4) Coping with loneliness. The loss of in-person interaction contributed to feelings of loneliness and digital interaction was viewed as an insufficient alternative. Social freedom could be constrained by distancing policies and by social contacts, contributing to strained personal relationships and feelings of frustration as part of loneliness. Fluctuations in mood and difficult emotions were experienced alongside loneliness, and distraction and seeking reconnection were commonly reported methods of coping, although they were less accessible. These findings indicate that physical distancing measures can impact loneliness due to the limitations they impose on in-person social contact and the perceived insufficiency of digital contact as a substitute.

## Introduction

Loneliness is a distressing emotional experience which is highly relevant during the coronavirus disease 2019 (COVID-19) pandemic. The pandemic has necessitated physical distancing measures which compel people to avoid gathering with others as much as possible to limit viral spread. This vastly decreases opportunities for social contact at a time when the general public are concerned about the impact of the pandemic on isolation, mental health, and relationships [1]. Researchers have echoed concerns about the effects of the pandemic on mental health, with loneliness being implicated in this context [2, 3]. Evidently, physical distancing policies can be effective in flattening the curve of the pandemic [4] and are necessary to limit the spread of COVID-19. However, they markedly disrupt normal patterns of social interaction, potentially impacting social connection and loneliness. Therefore, it is vital to understand and consider the social and psychological effects of these COVID-19 disease containment policies. This will heighten our understanding of the psychosocial impact of lockdown and may inform the development of supports to mitigate potential negative outcomes in response to subsequent lockdowns and future pandemics.

Social isolation describes an objective situation in which social interactions are lacking [5], while loneliness is a subjective experience that arises when there is a discrepancy between the number or quality of social relations a person desires and the relations they actually achieve [6]. Loneliness has been found to predict poor physical and mental health [7, 8]. While social isolation may be ascertained via markers such as living alone and having infrequent social contact [9], loneliness must be described in terms of the feelings it entails such as emptiness and social pain [10]. Social isolation and loneliness are often associated with one another [11, 12]. However, they can also occur independently [13, 14]. A qualitative study by Cela and Fokkema [15] found that older Albanian and Moroccan migrants in Italy felt protected from social isolation by close family relationships but experienced loneliness because they lacked non-family peers to chat, reminisce, and discuss intimate issues with. Physical distancing and lockdowns related to COVID-19, which limit in-person interaction, may introduce circumstances akin to social isolation and affect loneliness in turn.

These measures have been described as “social distancing”, but the “physical distancing” description has been encouraged given that social interaction is still possible while people stay physically apart. Many people live with others who can provide social interaction. Depending on the restrictions in place, socialising with a limited number of others while keeping distance is also possible, for example by socialising outside. Digital means of interaction via phone and video calls, messaging, and social media are other potential modes of social connection. However, all of these forms of interaction depend on the resources available to an individual. The first is not available to those living alone, while the latter two depend on physical resources: access to a private outdoor space, nearby contacts, and access to a telephone or the internet. One in eight households in the UK does not have an outdoor space, with lower access among members of minority ethnic groups and those who are unemployed or casual, semi-skilled, or unskilled manual workers [16]. It is also important to consider the digital divide, which is the gap between people who do and do not have access to information and communications technology [17]. This can be driven by physical, motivational, and proficiency factors [17]. The digital divide is one way in which existing sources of social inequality are enacted [18, 19]. In the UK, people who are older, disabled, women, economically inactive, and from low-income households are more likely to be internet non-users [20]. Clearly, then, there are inequalities in potential access to social contact while practising distancing. Additionally, some individuals need to adhere to stricter, longer-term distancing or self-isolation if they contract or may have contracted the virus or have a health condition that makes them particularly vulnerable [21]. However, in-person interactions are globally reduced by physical distancing policies, introducing a period of “enforced social isolation” [22, p. 5] for all members of the population adhering to these guidelines.

Even for those who can socialise using digital means, there may be discrepancies between face-to-face and digital social interaction which could affect personal relationships. In a cohort study of adolescents, Twenge and colleagues [23] found that time socialising in-person has decreased since the 1980s as digital options for interacting have increased. Importantly, adolescents who reported little in-person interaction and high social media usage were found to be loneliest, suggesting that social media and in-person interaction may not equally mitigate loneliness. In-person interaction has been found to be associated with higher positive mood than interaction via text messaging in times of stress [24]. Moreover, in-person interactions were found to be more satisfying and to entail higher self-disclosure and closeness than interaction over internet chat messaging [25].

There is a dearth of qualitative research relating to the COVID-19 pandemic. Vindrola-Padros and colleagues [26] have highlighted how timely qualitative data can uncover aspects of the pandemic experience that are missed by clinical and epidemiological studies. It appears that loneliness may not have risen during the first phase of COVID-19 lockdowns [27–29], although 13.8% of US adults indicated that they were often or always lonely in an April 2020 measurement [29] and 27% of UK adults were found to be lonely in the first month of lockdown [30]. The comparatively small amount of qualitative research also indicates that loneliness is a concern. A qualitative survey of US adolescents included loneliness as one of the reported mental health challenges [31]. Additionally, a survey and focus group study of 11- to 25-year-olds in lockdown in Northern Ireland found that 45% of respondents reported loneliness and isolation [32]. Moreover, a mixed-methods study of US older adults found that those who reported worsening loneliness during shelter-in-place orders described physical and mental health impacts and feelings of being trapped [33]. The effect of loneliness has often been highlighted in research with older adults and clinical populations. In studying the impact of COVID-19 and related public health measures on UK adults with eating disorders, Brown and colleagues [34] described social isolation as a key theme, with loneliness mentioned in all of ten in-depth interviews. Clearly, social isolation and loneliness are a concern during the COVID-19 pandemic and associated physical distancing and lockdown measures. Given that loneliness has been found to be prevalent across the lifespan and particularly in younger and older adults [35], a life-course approach which examines loneliness in individuals at different ages is appropriate. However, no study, to our knowledge, has specifically explored experiences of loneliness in the context of the COVID-19 lockdown using qualitative methods.

To address this gap, this study explored the following research question: What are people’s experiences of loneliness while practising physical distancing due to a global pandemic? This study is contextualised within the initial COVID-19 lockdown in the UK which began on March 23^rd^, 2020. These measures came into place twelve days after the outbreak was designated as a global pandemic by the World Health Organisation and compelled people to stay at home with the exception of medical or essential work purposes, shopping for necessities, and taking exercise once a day. In the week prior to March 23^rd^, 2020, the UK confirmed 4.38 COVID-19 deaths per million people, relative to 64.92 in Italy and 0.80 in the Republic of Ireland [36]. At the end of May, this had risen to 21.82 per million in the UK [36].

## Method

### Design

Semi-structured telephone and videoconferencing interviews were carried out to explore experiences of loneliness, isolation, and social contact among people practising physical distancing during the initial COVID-19 lockdown in the UK. Interviews were utilised as they can uncover valuable knowledge about participants’ experiences [37]. The consolidated criteria for reporting qualitative research checklist (COREQ) guided the reporting of this research [38]. Ethical approval was granted by the Research Ethics Committee in the Faculty of Engineering and Physical Sciences at Queen’s University Belfast on April 9th, 2020.

### Methodological approach

This research was underpinned by an ontology of critical realism, wherein the social world is believed to filter what we can discover about a potential reality; in this way, “ontological existence does not necessitate epistemological awareness” [39, p. 2]. We can aim to identify and describe loneliness during physical distancing without assuming that we are accessing the full breadth of the phenomena. Participants’ accounts of loneliness are accepted as having an experiential reality but not regarded as independent from their context [40]. Epistemologically, the research is grounded in contextualism, with all knowledge considered as situated [41]. Qualitative results are not seen as offering complete, universal truths, but it is believed that they can be valid when grounded in data [42].

Reflexive thematic analysis was chosen to analyse the data. Thematic analysis is a flexible method for identifying themes and patterns in data which can be used to answer research questions about experiences [43], which is the aim of this study.

A reflexive thematic analysis approach was utilised in which the researcher’s role is considered and acknowledged [44]. The themes were generated through continual, reflexive engagement with the data through the process of analysis. This took a primarily experiential approach in line with the critical realist frame of this research [45]. The analysis was grounded in the data rather than overarching theory.

### Participants

Participants were purposively sampled and primarily recruited via an online survey of adults practising physical distancing and not working outside the home (unpublished work). The survey was advertised through researchers’ networks and social media platforms via posts describing the research study on Facebook and Twitter. Individuals who had felt lonely during physical distancing were sought via recruitment materials asking for participants to take part in an interview to discuss their experiences of loneliness in this context. Survey respondents who reported that they had been lonely all of the time or often in the past week were invited to provide an email address if they lived in the UK and were willing to take part in an interview about their experience of loneliness during physical distancing. Of fifty eligible survey respondents, ten provided an email address and seven responded to an email inviting them to interview. As these participants were all women, a call was additionally disseminated through researchers’ networks and social media to recruit men who had felt lonely during physical distancing. Two participants were recruited through this call. Eight participants were interviewed and included in analysis. A ninth interviewee was not included in analysis as they did not fulfil the inclusion criteria. The inclusion criteria were: being over the age of 16, practising physical distancing, living in the UK, not currently working outside the home (i.e. an essential worker) or living in a residential care setting, not self-isolating or quarantining due to symptoms of COVID-19 or feeling physically unwell, and not currently receiving treatment for a significant ongoing mental health condition.

Following these interviews, a discussion between two members of the research team (PMP and JG) reflected on the data and whether additional participants should be interviewed. Ultimately, this number of participants was deemed sufficient to access rich, multi-faceted information given the richness of the data and scope of the project. This sampling strategy follows Braun and Clarke’s [46] assessment that data saturation is not a particularly useful concept for reflexive thematic analysis and pragmatic decisions about richness and scope might better guide sampling decisions. A summary of participant characteristics is presented in Table 1. Of these, four were working remotely, two were students, one was retired, and one had recently lost their job.

**Table 1:** Interview participant characteristics.

Participant ID	Gender	Age	Location	Interview Medium	Living Situation
1	Female	27	Northern Ireland	Telephone	Living alone
2	Female	21	Northern Ireland	Telephone	Living with family members
3	Female	37	Northern Ireland	Microsoft Teams	Living with family member
4	Female	30	England	Telephone	Living with housemate (non-family)
5	Female	24	England	Telephone	Living with housemates (non-family)
6	Male	57	England	Zoom	Living with family members
7	Female	67	Northern Ireland	Telephone	Living with partner
8	Male	48	Northern Ireland	Telephone	Living alone

### Procedure

Participants were contacted via email and invited to take part in an interview over the telephone or Skype. In this email, they were invited to complete an online written consent form hosted on Qualtrics [47]. Two participants indicated that they would like to use Microsoft Teams and Zoom, so these platforms were used. Each participant was interviewed on one occasion in May, 2020. The longest interview lasted 46 minutes and the shortest 26 minutes, with an average length of 35 minutes.

The interview guide was developed by JG and PMP and designed to elicit information regarding experiences of loneliness, social isolation, social contact, and methods of coping during physical distancing (see S2 File). A number of probes were included to encourage participants to provide more detail where it could be helpful [43]. The interview guide was considered flexible such that all topics could be covered but a set structure did not have to be followed, allowing topics to be addressed in a different order if participants raised them spontaneously. Prior to the interview, the interviewer (PMP) introduced herself and engaged in small talk to build rapport. Participants were informed that the study aimed to understand more about experiences of loneliness and physical distancing. Participants were invited to ask any questions and asked if they were comfortable with the interview being recorded (all consented). A Zoom handheld audio recorder was used. After covering the interview topics, participants were asked if there was anything they felt the interviewer had left out. Following the interview, participants were thanked for taking part and sent a list of resources for support organisations (e.g. the Samaritans). Initial impressions were noted by the interviewer.

### Reflexivity

The interviewer (PMP) was a female Master’s student with a BA in Psychology and advanced training in qualitative methods and interviewing. PMP had no prior relationship with the participants. Throughout interviewing and analysis, PMP endeavoured to remain aware of personally held assumptions and judgements about loneliness and the circumstances that may or may not be associated with it, such as living alone, during physical distancing, and to avoid imposing these on analysis. While researcher and context may inevitably affect analysis [48], this was deemed important to remain grounded in the data rather than imposing an unintentional, unfounded framework.

### Analysis

The interviews were transcribed verbatim with all words, sounds such as sighs, and major paralinguistic features recorded. Transcripts were entered into NVivo 12 software [49].

The process of reflexive thematic analysis [44] involved initial repeated reading of transcripts for familiarisation with early codes and points of interest noted. The data were then fully coded so that all extracts which could be relevant to the research question were given a label. Codes were reviewed to check that they were coherent and well labelled and were then arranged into potential themes derived from the data which captured important, relevant patterns. These candidate themes were reviewed and developed for coherency, distinctiveness, and good representation of the data. Following this, the interviews were reread to explore whether the themes represented the dataset well. This resulted in changes, such as the promotion of one subtheme to a full theme after reflection on its relevance and distinctiveness. Coding and candidate theme development were undertaken by the primary researcher (PMP), with a second researcher (JG) coding two randomly selected transcripts to allow different perspectives. The two codings were found to be largely similar. Following initial theme development, the themes were developed and refined in collaboration with the whole research team. Transcripts and findings were not returned to participants following analysis.

### Doing research digitally

The pandemic affected the way that data could be collected. Digital means of recruitment and data collection were embraced from the outset of this research. While remote methods (i.e. phone and video calling) have often been presented as inferior to face-to-face interviewing, this perception may be unfounded [50]. In practice, they present various strengths such as scheduling flexibility and the ability for participants to choose a comfortable, private setting [51]. Remote interviews have also been described as a useful forum for asking sensitive questions and facilitating less inhibited responses [52].

## Findings

Four main themes were generated in analysing participants’ accounts of loneliness and isolation during physical distancing (see Table 2).

**Table 2:** Themes and subthemes.

Themes	Subthemes	Illustrative Quotation
1. Loss of In-Person Interaction Causing Loneliness		“You sort of don’t realise how sociable you are, or how much of a routine you’re in of seeing people until you physically can’t.” (Participant 2, 21-year-old female)
	1.1. Digital Interaction as Inferior	“You don’t have that visual cue of ‘Am I going too far, can–are they okay if I say this?’” (Participant 2, 21-year-old female)
	1.2. Missing Touch	“Not hugging my parents […] is weird.” (Participant 1, 27-year-old female)
2. Constrained Freedom		“The curtailment of freedom is the issue.” (Participant 3, 37-year-old female)
	2.1. Strained Relationships	“The father’s a bit anxious that the kids […] don’t come into contact so […] if she sent the kids here to us then he’d find out and he’d give her hell to pay.” (Participant 6, 57-year-old male)
	2.2. Frustration	“It wouldn’t have that like sort of frustration element to it. I think it’s because we, we physically don’t have…like, the choice.” (Participant 2, 21-year-old female)
3. Challenging Emotions		“I’ve had days and sometimes longer when I felt really frustrated and really down.” (Participant 7, 67-year-old female)
	3.1. Ups and Downs	“Ups and downs, am, good days, bad days.” (Participant 8, 48-year-old male)
4. Coping with Loneliness		“I think I have like quite like a decent understanding of how I feel generally and like how to make myself feel better but […] there’s not so many options for that.” (Participant 5, 24-year-old female)
	4.1. Seeking Reconnection	“That’s a little bit more difficult at the moment but […] like planning phone calls with other people.” (Participant 4, 30-year-old female)
	4.2. Aiming for Distraction	“I just sort of don’t think about it and distract myself.” (Participant 1, 27-year-old female)

### 1. Loss of in-person interaction causing loneliness

Loneliness while physical distancing was experienced as a consequence of the loss of in-person interaction and the related inferiority of digital contact and lack of physical touch. Several participants reported the loss of in-person interaction as causing feelings of loneliness. This was noted in several ways. In describing what caused loneliness during physical distancing, the lack of in-person interaction was consistently raised.

“I think it’s probably the not socialising like face-to-face.”

(Participant 1, 27-year-old female)

Participants mentioned loneliness in relation to missing specific people and the social support they provide when socialising in-person, as well as the inability to passively be around others, for example by spending time in a busy library or café. The sense of social presence was lacking as participants could not casually spend time around others. Physical distancing had disrupted the normal routine of seeing others, leading to loneliness and a lack of distractions.

“You sort of don’t realise how sociable you are, or how much of a routine you’re in of seeing people until you physically can’t.”

(Participant 2, 21-year-old female)

Some participants indicated that being used to social isolation could potentially make the experience of physical distancing easier. This was noted by one participant who lived rurally, another who had been through a previous isolating experience of living with someone with a substance use problem, and one who suggested that someone in their life found the situation more manageable by virtue of being a generally less sociable person.

Two subthemes were relevant to this theme: ‘Digital Interaction as Inferior’ and ‘Missing Touch’.

#### 1.1. Digital interaction as inferior

Digital interaction via video calling, phone calls, messaging, and social media were consistently described as “no comparison” to in-person interaction and portrayed as a means to an end during physical distancing and “not a long-term solution” (Participant 8, 48-year-old male). This was represented in terms of a somewhat intangible difference between in-person and digital interactions, where in-person interaction is “just real, it’s more real” (Participant 1, 27-year-old female), containing a certain energy or atmosphere that is lacking in digital interactions.

“You felt quite isolated because it’s not the same doing it on a phone call or doing it on a video chat or doing it in a text message.”

(Participant 3, 37-year-old female)

However, this was also related to objective limitations of digital interaction, such as the presence of other people hindering openness, technical issues, or a disparity in the use or preference of certain platforms by social contacts. The ability to comprehend and use technology was mentioned by several participants as a facilitator or barrier to connection through digital means. Several participants also mentioned the lack of visual cues as a limitation to digital interactions. This was described even in the context of video calls. Without a detailed understanding of others’ body language, it was harder to read the other person because “you can’t gauge people properly on the other end of a computer” (Participant 7, 67-year-old female). Only one participant described a digital interaction as comparing well to in-person interactions with respect to the openness of a phone call they had enjoyed with a friend.

#### 1.2. Missing touch

The lack of physical touch was referred to by several participants of varying ages and genders as strange and something that they missed. In reflecting on how they missed physical contact with others, one participant who was living alone reported not having touched another person for over two months, which was “very weird” (Participant 8, 48-year-old male).

“I think I really miss having like physical contact with other people, […] it’s just not really normal to not touch anyone.”

(Participant 5, 24-year-old female)

Two participants specifically mentioned not being able to hug their loved ones in the context of bereavement, which was described as “really bizarre” (Participant 4, 30-year-old female). It was difficult not to hug loved ones who were crying, but it could also simply be awkward not being able to touch others during regular interactions.

### 2. Constrained freedom

The experience of loneliness in this context was linked to a sense of constrained freedom which could strain relationships with others and introduce an element of frustration into feelings of loneliness. While feeling lonely and missing in-person interaction, participants also expressed frustration at what they perceived to be a lack of agency and choice over their social lives during the first phase of lockdown. Distancing restrictions played a role in loneliness because they constricted participants’ ability to connect with others. Knowing that they couldn’t socialise was difficult.

“The curtailment of freedom is the issue […] so like, I’d be quite happy to stay in the house and watch TV for a weekend instead of going out but now it’s–I can’t, I don’t have that choice is more the problem.”

(Participant 3, 37-year-old female)

Freedom was constrained both by government rules around lockdown and by the preferences and fears of others. The government guidance was depicted by some as overlooking people living in certain situations, such as young people outside of nuclear families or those living rurally who might need to travel further to see others and therefore lack the casual social connection of seeing neighbours. This oversight was described as “a huge intrusion into people’s personal lives” (Participant 5, 24-year-old female).

Two subthemes were relevant: ‘Strained Relationships’ and ‘Frustration’.

#### 2.1. Strained relationships

The constrained freedom of physical distancing could strain personal relationships. The ability to socialise in-person could be hindered by others’ preferences about socialising during the pandemic, so arguments could be caused by face-to-face distanced socialising if others did not feel that this was safe or in their best interests. One participant described an argument with her housemate due to their different views on face-to-face distanced socialising which made her hesitant to socialise again despite her desire to do so. Living by their rules made her feel “sort of disempowered in that sense, like I don’t […] have that much agency or independence.” (Participant 4, 30-year-old female). Strain could also be caused by being forced to spend so much time with others in the household, although this could eventually level out.

“Tensions kind of run high when you’re not used to being permanently together.”

(Participant 2, 21-year-old female)

One participant also described their loneliness as morphing into frustration and anger at their partner who did not find this restriction equally difficult, which could lead to them expressing anger at the partner.

#### 2.2. Frustration

In the context of constrained freedom, participants also expressed frustration within their descriptions of loneliness during physical distancing. This frustration came about because distancing measures acted as an uncontrollable block to seeing others. During distancing, loneliness could contain an element of frustration about not being able to see other people due to the restrictions. Compared to lockdown loneliness, previous experiences of loneliness “wouldn’t have that like sort of frustration element to it” (Participant 2, 21-year-old female). Participants also expressed frustration at the nature of life during physical distancing, which was described as repetitive and less busy.

“There’s been a real Groundhog Day sense every now and again so there has been a, there has been a exasperation. A like a (sighs) ‘Jesus, here we go again.’”

(Participant 6, 57-year-old male)

### 3. Challenging emotions

In lockdown, loneliness was experienced in concert with other challenging emotions and would fluctuate alongside these difficult feelings. As well as frustration at the loneliness and social block created by distancing, most participants described a number of challenging emotions related to loneliness and isolation during physical distancing. These negative affective experiences included boredom, worry, fear, sadness, anger, and anxiety. Some participants expressed a sense that these feelings were heightened during physical distancing restrictions. Although these feelings might not be new to participants, they hadn’t been felt “to the same extent” (Participant 2, 21-year-old female) outside of distancing.

“I feel like my baseline anxiety is just like quite high all the time at the moment […] it’s higher on some days and not on other days, I can’t really, um…I just trust that it will be better, um, you know the–the feeling will pass.”

(Participant 4, 30-year-old female)

These emotions were often described as coinciding, such that a situation was both upsetting and frustrating, or a participant would feel isolated and depressed on a given day. The feeling of loneliness was also described as entailing guilt when it occurred despite the availability of social contacts. Participants recognised that challenging emotions might be widely experienced during lockdown. Some would discuss these feelings with friends or family, while others suggested barriers despite the shared experience. This was situated as a way to avoid complaining or forcing their bad mood onto the people close to them who might already be distressed or, in contrast, not finding the situation distressing.

One subtheme was identified as relevant.

#### 3.1. Ups and downs

Physical distancing was consistently described as entailing fluctuations in mood and loneliness which could occur without a clear reason. Some days were fine, and participants felt that they could handle distancing, while on others they experienced difficult emotions. Loneliness would occur in “hits” (Participant 3, 37-year-old female) or “waves” (Participant 1, 27-year-old female) which were often unpredictable. This was also the case for other difficult feelings.

“There’s been days where I’ve felt very kind of isolated and depressed and then there’s other days when I’ve felt in really good form […] there hasn’t seemed to be, uh, any kind of reason, rhyme or reason to it particularly.”

(Participant 8, 48-year-old male)

### 4. Coping with loneliness

The experience of loneliness, other challenging emotions, and lack of agency during distancing prompted attempts to cope via methods such as seeking reconnection and distraction, which could be challenging in this context. Each participant described mechanisms to attempt to cope with physical distancing or loneliness specifically. Some participants appeared to attempt to cope by practising perspective-taking and recognising that others were experiencing the same circumstances. Indeed, some participants directly invoked the universality of their current feelings when describing how they felt. The difficult feelings associated with distancing were not portrayed as specific to the individual, given that in lockdown everyone was “in the same boat” (Participant 1, 27-year-old female).

Overall, it appeared that seeking to reconnect with others and aiming to distract oneself were the main methods that participants used to cope with loneliness and low mood, and physical exercise was also commonly mentioned with respect to coping with physical distancing.

Two subthemes were relevant to this theme: ‘Seeking Reconnection’ and ‘Aiming for Distraction’.

#### 4.1. Seeking reconnection

Most participants mentioned reaching out to connect with others as a common and effective means of alleviating loneliness. This drive to reconnect purely to alleviate loneliness was portrayed clearly.

“I’ll phone my mum or something and ask her a really stupid question […] like, ‘If I defrost this, can I…?’ or ‘Can I cook this from frozen?’, even though I’ll know.”

(Participant 1, 27-year-old female)

Outside of physical distancing, participants mentioned that they might plan to meet up with a friend or work in a space with others, while in the context of physical distancing they would digitally contact friends or family, spend time with someone in their home, or plan digital contact with others. However, physical distancing acted as a barrier to reconnection, offering “less opportunities to actually go and meet people” (Participant 8, 48-year-old male).

#### 4.2. Aiming for distraction

Participants consistently described trying to keep busy and distract themselves to cope with feelings around physical distancing. However, this was made more difficult by a perceived lack of distractions during physical distancing.

“You can’t really go out and do things to distract yourself and you can’t see people to distract yourself.”

(Participant 5, 24-year-old female)

Participants described various avenues to distraction within the confines of physical distancing, with various hobbies including physical exercise, reading, and watching television commonly mentioned. These distractions functioned as ways to manage feelings of loneliness, keep busy, balance mood, and relax.

“If I go out to the garden or just start to do something with plants, that makes me feel a lot better so I can lose those feelings of isolation and loneliness because I feel I’m doing something worthwhile.”

(Participant 7, 67-year-old female)

## Discussion

The aim of this study was to understand how people experienced loneliness while physical distancing during the initial COVID-19 lockdown in the UK. This is an important context for studying loneliness given that it is generally unprecedented and impacts the availability and means of social contact. Qualitative analysis of semi-structured interviews with adults in Northern Ireland and England produced four themes relevant to this research question: (1) Loss of in-person interaction causing loneliness, (2) Constrained freedom, (3) Challenging emotions, and (4) Coping with loneliness. Individuals in various age groups and living situations were interviewed, allowing the findings to shed light on a range of developmental and environmental contexts in which loneliness was felt. In doing so, this is the first qualitative study to focus explicitly on experiences of loneliness during lockdown.

The present findings indicate that the loss of in-person interaction contributed to loneliness during lockdown, despite findings suggesting stable levels of loneliness during the pandemic [28, 30]. This disruption may play a key role in lockdown loneliness, given that some participants described the protective nature of previous isolating experiences. Being used to social isolation, for example as a result of living in an isolated area, was reported as making physical distancing less challenging. While loneliness may not be generally rising during the COVID-19 pandemic, it appears that the loss of in-person interaction can nevertheless impact loneliness. Indeed, focus group research conducted in the first two weeks of UK lockdown found that the loss of in-person social interaction could lead to psychological and emotional loss for some people [53]. This study indicates that loneliness can be an outcome of this loss for individuals of various ages.

The loneliness described by participants also captures the perceived inferiority of digital social interaction relative to in-person interactions. There has been momentum behind the use of “physical distancing” terminology [e.g. 54], as socialising is still possible under these conditions. In light of this, it is compelling that participants in this study positioned digital interaction as an insufficient alternative. Even video calls were described as inadequate at construing the visual cues that make it possible to read others and gauge interactions. This aligns to some degree with experimental research finding that social support provided via text messaging does not improve mood as much as in-person support in times of stress [24] and that higher closeness and self-disclosure are experienced in face-to-face interactions [25]. Overall, a picture emerged in which digital contact is a key way to socially connect during the pandemic but is a means to an end rather than a satisfactory alternative to in-person interaction. Moreover, some individuals have less access to digital contact due to a lack of access to the internet or internet-enabled devices, poor internet connection, or having social contacts who lack access. Even among those who do, digital contact appears to be less satisfactory. The role of discrepancies in digital utilisation on social contact during distancing was also partly visible through this research. Participants described how others use or disuse of digital technologies affected their ability to interact during lockdown. While participants were using these technologies, their friends and family were not necessarily doing so. Some participants mentioned how the ability to fluently use digital platforms–others’ “technological awareness”–affected whether they could connect this way. It appears that this lack of digital access and fluency can affect social connection as reported by people who do utilise these technologies. The perceived inadequacy of digital contact is important as distancing measures remain in place and subsequent regional and national lockdowns have occurred since these data were collected, making digital communication the safest available option for many people. Increasing digital access and proficiency, enhancing the quality and satisfactoriness of digital contact, and enabling safe face-to-face contact may therefore be key methods for limiting loneliness.

The lack of physical touch was relevant to this driver of loneliness. Participants described the irregularity of not being able to touch other people, which had spanned months for one individual. Participants also raised the strangeness of being unable to provide supportive touch in the context of bereavement. These circumstances are affecting many people as COVID-19 is a mass bereavement event [55], having caused over a million deaths worldwide [56]. Moreover, touch has been implicated in regulating the physiological response to acute stressors and re-establishing social contact after time apart [57, 58], both of which are highly relevant in this context. It appears that touch is missed and implicated in loneliness at this time when it is necessarily limited.

Loneliness was also impacted by government restrictions around socialising in person, as well as by other people, leading to perceptions of constrained freedom. Personal agency allows individuals to affect their environments and lives, with implications for self-efficacy and wellbeing [59, 60]. The change in social agency may impact loneliness and wellbeing in this context. For participants living in households without friends, family, and partners or in geographically isolated areas, the lockdown may have a larger impact, and some reported feeling unfairly impacted by government restrictions. This may appear to run counter to the findings of Bu and colleagues [61] that living in a rural area was protective against loneliness during the initial UK lockdown. However, participants mentioned that previous experience of isolation, for example due to living in an isolated area, could limit the impact of distancing on loneliness. The constraints of lockdown also strained relationships by forcing households to spend an unusually large amount of time together. There could be interpersonal tension and further constraint when views about safe or sensible socialising did not align with people around an individual (e.g. if a friend does not want to socialise face-to-face) and others (e.g. if third parties, such as the relatives of a housemate, do not want the individual to meet others). Prosser and colleagues [62] note that interpersonal differences are likely to become more salient as regulations relax, with some individuals continuing to practise stringent physical distancing and others not. These different approaches appear to affect individuals’ ability to exercise social agency and affect interpersonal relationships, and can therefore impact loneliness.

As a result of the constraints associated with lockdown, there was a feeling of frustration as part of loneliness that was not present in previous loneliness experiences. Distancing measures blocked participants’ ability to socialise in person, entailing a dual sense of loneliness and frustration. This is worthy of careful consideration, given that frustration has been associated with low adherence in other contexts, such as physical therapy [63]. Moreover, Ripper and colleagues [63] note that making individuals aware of potential frustration and psychological difficulties may improve adherence. Although physical distancing is markedly different, public awareness of the potential for frustration and ways to manage it may be beneficial to encourage adherence to disease-containment guidance. Furthermore, recent research found that socialising to avoid loneliness was a key reason for not complying with distancing rules [64]. The present findings should be considered with respect to their implications for physical distancing adherence given that it is a vital means of controlling the spread of COVID-19.

Beyond the frustration that characterised lockdown loneliness, participants also described physical distancing as a time of fluctuating emotional challenges. Loneliness and feelings such as anxiety and boredom would occur in waves with unpredictable good and bad days. This echoes a participant of Brown and colleagues [34] who mentioned that loneliness originally occurred in bursts but became more painful as lockdown continued. In studying loneliness from childhood to young adulthood, Rönkä and colleagues [65] noted that the intensity of loneliness could fluctuate even when it lasted several years. However, as Palmer [66] points out, most quantitative loneliness measures do not assess fluctuations, leaving this aspect of loneliness relatively untapped. It also appears that loneliness may occur alongside other difficult emotions in lockdown. Indeed, Groarke and colleagues [67] found that loneliness was associated with subsequent depression and vice versa during lockdown.

These findings additionally shed light on the nature of coping during physical distancing. The main strategies described were seeking reconnection with others and distraction. It has been theorised that there is a natural drive to reconnect with others in the face of loneliness [68]. Similarly, qualitative work with lonely university students found that support-seeking was common and social connection was considered valuable when loneliness struck [69]. The use of distraction to cope with loneliness has also been identified in lonely university students and older adults [70]. Encouragingly, meta-analytic work has shown that distraction is one of the most effective methods of managing negative feelings [71]. However, we found that these strategies were less accessible during physical distancing. Participants described fewer avenues for reconnection and distraction because they couldn’t go out and meet others. Although these forms of coping were less available, some participants additionally appeared to practise perspective-taking, recognising that the difficulties experienced were somewhat universal. This may allow lonely individuals to feel kinship with others despite the limited social options. These findings add weight to research and public concerns about social and psychological wellbeing during the pandemic [1, 3, 72].

### Strengths and limitations

The reliance on online methods is a limitation of this study, making it difficult to recruit individuals without internet access, who may be more affected by the social impact of lockdown. While there are a number of advantages of online research, the digital divide is relevant here. The discrepancy in internet access, use, and proficiency excludes people from research conducted online. While the pandemic circumstances necessitated the use of online methods in this study and the vast majority of others at this time, the potential for exclusion is an important limitation. Moreover, while the sample included a wide age range of adults in England and Northern Ireland, participants in Scotland and Wales were not represented, and it is not clear how representative the sample were with respect to factors such as socioeconomic or migrant status, which can be related to loneliness [15, 61]. However, these findings are novel in shedding light on the experience of loneliness, a highly prevalent and deleterious emotion, during the first phase of the COVID-19 lockdown in UK adults. Future research might expand on these findings by examining loneliness in subsequent phases of lockdown when individuals may have acclimatised to socialising in these circumstances. It may be the case that the findings would be altered by factors such as familiarity with the pandemic circumstances and changes to physical distancing and lockdown measures if the study were conducted later in the pandemic. It is also vital that future qualitative and quantitative work considers individuals affected by the digital divide who are less able to safely access social interaction at this time.

## Conclusion

The present study sheds light on the experience of loneliness while physical distancing in the initial UK COVID-19 lockdown. The findings suggest that the loss of in-person interaction contributes to loneliness and digital interaction is an insufficient alternative. Physical distancing imparts constraints on socialising which may be particularly salient for some individuals, such as those living separately from friends and family or those who are geographically isolated. Strained personal relationships and a number of challenging emotions were experienced alongside loneliness, while key coping methods were less available due to the restrictions. Given the likelihood of ongoing lockdowns and future pandemics, it is vital to consider loneliness in these circumstances, both in the general population and in specifically affected groups such as people living alone or apart from loved ones, geographically isolated individuals, and those affected by the digital divide.

## Supporting information

S1 File COREQ checklist. (DOCX)

S2 File Interview guide. (DOCX)
