# Supplementary material for: Connection, constraint, and coping: A qualitative study of experiences of loneliness during the COVID-19 lockdown in the UK
Source: PLoS One. 2021 Oct 13;16(10):e0258344. doi: 10.1371/journal.pone.0258344 (PMC8513854; doi:10.1371/journal.pone.0258344)
Supplement: S2 File — (DOCX) [file pone.0258344.s002.docx]

Supplementary Material:

Semi-structured Interview Guide

- Thank participant for agreeing to take part
- Introduce self

**Notes for interviewer:**

- As described in the Participant Information Sheet, we are interested in hearing about your experience of social distancing, as well as any feelings of loneliness you might have experienced recently.
- If at any time during the interview you do not wish to answer a question that’s okay.
- I would like to record our conversation. The recording will be typed out, but everything you say will be anonymous. Your name and any names or places you mention will be taken out, so that if someone read your interview they would not know who you are.
- If, at any stage, you wish to stop the audio recorder, please let me know.
- Do you have any questions?

1. **Social isolation**

- How have you been finding social distancing so far?
- What’s changed in your life compared to before the lockdown?
- Could you describe your social life during social distancing?
- How often have you been able to connect with others since social distancing measures were introduced? (e.g., physical and/or virtual)
  - Have you been using technology?
  - Have you been using social media?
    - Is that to connect with people, or looking at news feeds?
- Do you live with anybody else? (e.g. friends, family, partner)
  - Is that the same as your living situation outside of social distancing?
- Have you been spending much time alone lately?

1. **Social connection**
   - Tell me about your most recent social interaction
     - Was it face-to-face at a distance, virtual?
     - Was it enjoyable, satisfying, awkward?
     - How did it compare to your social interactions before the lockdown?
2. **Loneliness**

- Have you felt lonely since the lockdown began?
- How has loneliness felt for you?
- Tell me more about your most recent episode of loneliness
  - E.g., how did it feel?
  - What caused it?
  - How long did it last?
- How does that compare to times when you weren’t social distancing?

1. **Coping**

- Have you been doing anything to cope with social distancing?
  - Has that helped, do you think?
- Have you been doing anything to feel more socially connected?
  - Has that helped, do you think?
- Is there anything that has helped you feel less lonely?
  - Has that been making you feel more lonely or less lonely?

1. **Summary**
   - Was there anything I left out?
   - Is there anything else you would like to tell me?

- Okay then, I’ll turn off the recorder now.
- Thank participant
- Mention sending along support organisation details
